# Circulating tumor DNA analysis detects micrometastatic disease and predicts recurrence in a patient with colon cancer: A case report

**DOI:** 10.1097/MD.0000000000034330

**Published:** 2023-07-14

**Authors:** Jiamin Luo, Bo Zhou, Liang Zhao, Jing Yuan, Jinming Zhou, Lu Shen, Fang Li, Chengyuan Qian

**Affiliations:** a Department of Oncology, Daping Hospital, Army Medical University, Chongqing, China; b Department of Hepatobiliary Surgery, Daping Hospital, Army Medical University, Chongqing, China; c Department of Oncology, Chongqing Shifang Hospital, Chongqing, China; d Department of Radiology, Daping Hospital, Army Medical University, Chongqing, China; e GenePlus-Beijing, Beijing, China.

**Keywords:** circulating tumor DNA, ·colorectal cancer, disease recurrence, ·micrometastatic disease

## Abstract

Colorectal cancer (CRC) is one of the most prevalent and deadly cancers worldwide, and approximately 50% of patients with early-stage disease develop metastases. A critical limitation for successful management of CRC is early disease detection and identification of progression. Next-generation sequencing-based circulating tumor DNA (ctDNA) profiling has emerged as a promising biomarker for the assessment of minimal or molecular residual disease in CRC.

**Patient concerns:**

The patient was initially diagnosed with resectable CRC with uncertain small lung nodules.

**Diagnoses:**

The patient was diagnosed with sigmoid colon adenocarcinoma placed at 15 to 20 cm above the anal verge (ypT4N1R0). Lung nodules were found in the apical part of the upper lobe of the right lung and the dorsal segment of the lower lobe of the left lung.

**Interventions:**

The patient received systemic therapy and local treatment and plasma ctDNA-MRD detection was performed for monitoring the molecular disease status after surgery.

**Outcomes:**

The patient achieved a complete response after treatment. However, he presented with disease recurrence in liver lesions. The postoperative ctDNA detection suggested the possibility of micrometastatic pulmonary disease, and that was confirmed by follow-up examination. Serial ctDNA detection revealed disease relapse ahead of radiologic imaging by a lead time of 9 months. This case demonstrated the potential of ctDNA analysis to be a sensitive and specific tool for the detection of micrometastatic disease and prediction of recurrence.

## 1. Introduction

Colorectal cancer (CRC) is one of the most prevalent and deadly cancers worldwide, with an estimated 1.93 million new cases and 935,173 deaths.^[1]^ Given the advances in treatments for CRC of all stages, the survival time in CRC has been improving. However, survival rates are available for different stage. The 5-year survival rate of people with localized stage CRC is 90%, but drops to 14% for metastatic CRC (mCRC) in United States (https://www.cancer.net/cancer-types/colorectal-cancer/statistics). A critical limitation for successful management of CRC is early disease detection and identification of progression. Cancer progression arises from micrometastases or minimal residual disease (MRD) persisting after initial therapy.^[2]^ Currently, detection and effective treatment of MRD are still a clinical dilemma.

Circulating tumor DNA (ctDNA) was a variable and a generally small fraction of circulating cell-free DNA and was found to carry cancer-specific molecular alterations in blood plasma and other body fluids.^[2]^ Recent studies demonstrated that ctDNA has emerged as a diagnostic and prognostic biomarker in multiple cancers, including mCRC.^[3,4]^ The ctDNA profiles had high concordance with tissue-based assays,^[5]^ suggesting ctDNA is a viable alternative to tissue-based genotyping as a useful indicator for diagnosis and treatment. Additionally, ctDNA detection can be repeated during cancer monitoring more easily than tissue biopsy, and enables assessment of tumor variability and heterogeneity.

Here, we present a case who initial diagnosed with colon adenocarcinoma with small lung nodules (cTxN2Mx). The use of serial ctDNA monitoring facilitated the identification of micrometastatic pulmonary disease, and the assessment of risk for disease progression. We present the following article in accordance with the CARE reporting checklist.

## 2. Case presentation

**Figure 1.**
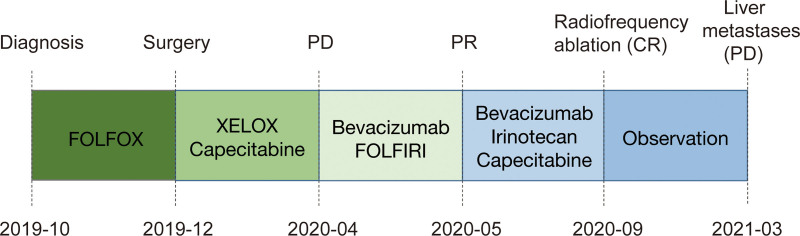
Clinical history of the patient.

**Figure 2.**
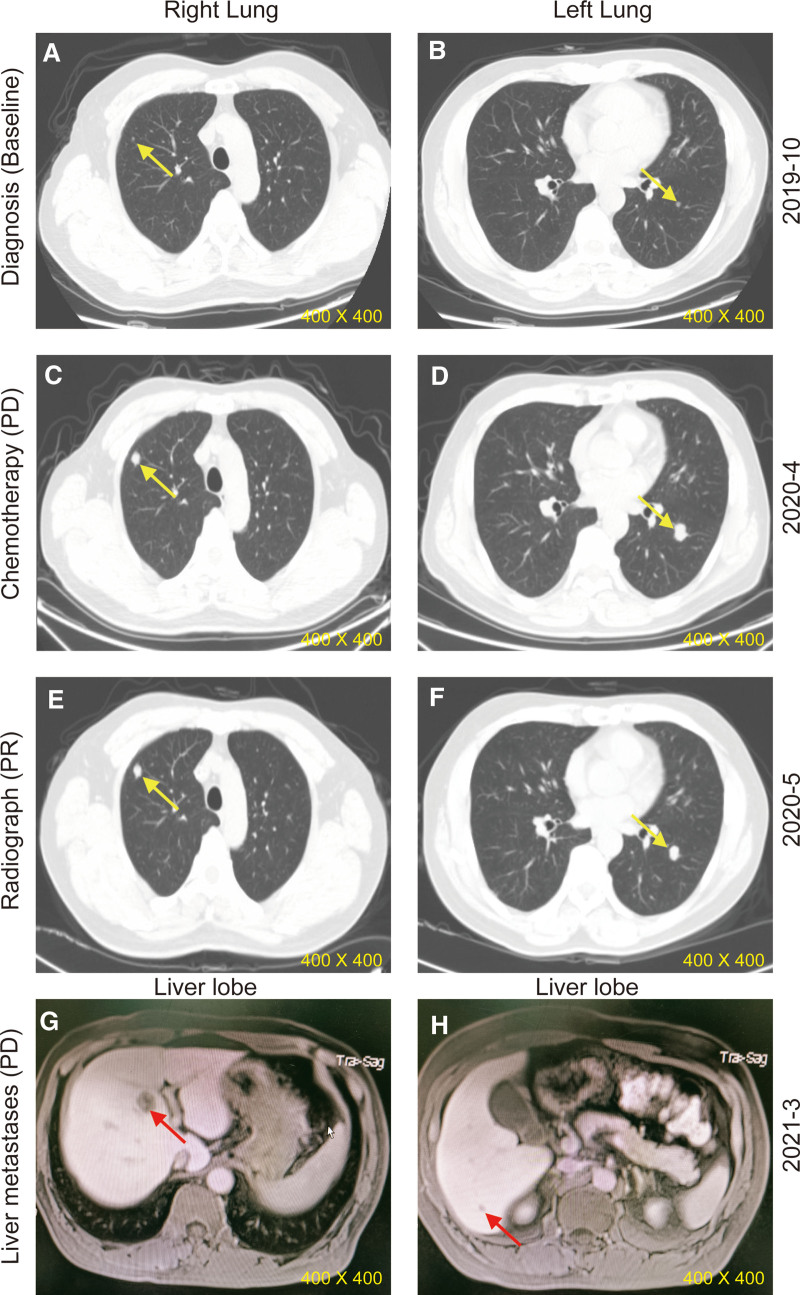
Image from chest computed tomography. (A) The apical part of the upper lobe of the right lung during diagnosis in October 2019; (B) the dorsal segment of the lower lobe of the left lung during diagnosis in October 2019; (C) the apical part of the upper lobe of the right lung during disease progression in April 2020; (D) the dorsal segment of the lower lobe of the left lung during disease progression in April 2020; (E) the apical part of the upper lobe of the right lung with PR after radiofrequency ablation in May 2020; (F) the dorsal segment of the lower lobe of the left lung with PR after radiofrequency ablation in May 2020; liver metastases on coronal (G) and axial (H) MRI images in March 2021. PR = partial response.

**Figure 3.**
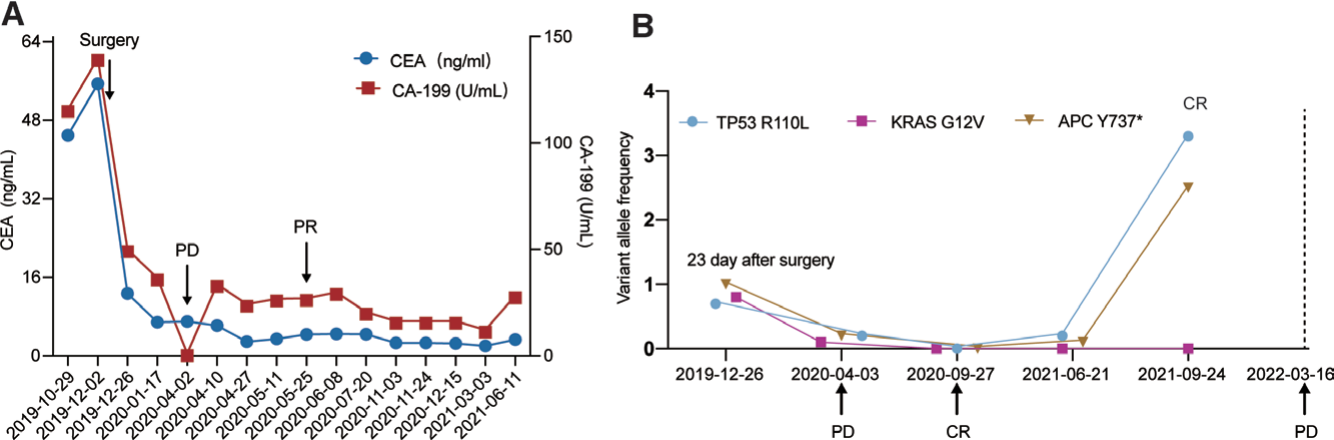
The levels of tumor marker and gene mutation during treatment in patient. (A) Dynamic changes of CEA and CA-199; (B) dynamic changes of genetic mutation fractions in ctDNA. CEA = carcinoembryonic antigen, ctDNA = circulating tumor DNA.

A 45-year-old man patient complained of persistent pain in the left lower abdomen, accompanied by a small amount of blood when defecating. He was diagnosed with sigmoid colon adenocarcinoma placed at 15 to 20 cm above the anal verge in October 2019 (Fig. 1). In addition, lung nodules were found by chest computed tomography (CT) in the apical part of the upper lobe of the right lung and the dorsal segment of the lower lobe of the left lung where the bigger one was 0.4 cm (Fig. 2A and B). The clinical stage was cTxN2Mx. At diagnosis the level of carcinoembryonic antigen (CEA) was 44.91 ng/mL (<5 ng/mL) and CA-199 114.78 U/mL (<37 U/mL) (Fig. 3A). Further testing identified a *KRAS* G12V mutation and microsatellite stable in his tumor, via capture-based targeted sequencing (1021 cancer-related genes).

Due to a skin allergy, the operation was delayed. The patients received a neo-adjuvant treatment with FOLFOX for one cycle and then he underwent a surgical resection of rectum and colorectal anastomosis (ypT4N1R0) in December 2019. Plasma ctDNA-MRD detection by tracking ultra-low-frequency somatic tumor mutations was used to monitor the patient’s molecular disease status after surgery and he was positive for ctDNA (Fig. 3B). Then the patient performed an adjuvant therapy with XELOX for 2 cycles from December 2019 to January 2020. Three cycles of maintenance therapy were conducted with capecitabine (1.5 g bid d1–14) in February 2020 (Fig. 1). However, CT scanning revealed a disease progression in April 2020 with an increase in the size of lung nodules (Fig. 2C and D), and they were ultimately considered as metastatic lesions.

Considering the *KRAS* G12V mutation, the patients received a combination therapy of bevacizumab plus FOLFIRI for 5 cycles. After 4 cycles of treatment, lung lesions were significantly decreased and radiograph revealed a partial response (PR) (Fig. 2E and F). The patient underwent radiofrequency ablation of lung lesions on August 24, 2020. One month later, CT scans showed a complete response (CR) in the lung lesions and he was negative for ctDNA (Fig. 3B). From November 2020 to April 2021, the patient subsequently received 3 cycles of maintenance therapy with bevacizumab plus irinotecan, followed by 5 cycles of capecitabine monotherapy. Notably, the results of ctDNA were positive again and the allele frequency of 2 tumor-specific mutations in APC and TP53 increased from June 2021 to September 2021 (Fig. 3B) but CT unremarkable. However, this patient presented with disease-recurrence 6 months later with liver metastases (Fig. 2G and H). This clinical time course is demonstrated in Figure 1.

All procedures performed in studies involving human participants were following the ethical standards of the institutional and/or national research committee(s) and with the Helsinki Declaration (as revised in 2013). Written informed consent was obtained from the patient to publish this case report and accompanying images. A copy of the written consent is available for review by the editorial office of this journal.

## 3. Discussion and conclusions

The presence of micrometastatic disease will ultimately determine the CRC-specific mortality of patients according to current guidelines. Despite development of continuous monitoring strategies utilizing advanced modalities (CT/MRI or PET-CT), or a repertoire of tumor biomarkers in blood (e.g., CEA, CA-199), detection of MRD or micro-recurrence, remains elusive. Emerging molecular liquid biopsies (e.g., ctDNA) provide a significant improved threshold for disease detection. In our case, small lung nodules were found at the diagnosis and it could not be confirmed that whether these nodules are normal pulmonary nodules or metastatic lesions due to imaging has identification limitations. While the analysis of ctDNA was positive at 23 days after surgery (Fig. 3B), suggesting the possibility of MRD or micrometastatic disease. Disease progression was detected in a CT exam with an increase in the size of lung nodules shortly after 3 months. These findings demonstrated the assessment of ctDNA in combination with improved imaging modalities may improve the prediction and identification of micrometastatic disease.

Furthermore, serial ctDNA monitoring provided a comprehensive view of the patient’s clinical and pathologic status, in a manner that is more precise than the methods currently used.^[6]^ The ctDNA detection could clarify equivocal imaging and/or CEA findings to enhance disease monitoring accuracy in gastric cancer.^[7]^ In stages II to III CRC, postoperative ctDNA inferred tumor recurrence ahead of radiological imaging.^[8]^ In our results, his ctDNA was positive at April 2020, which provided an indication of disease progression. On the other side, the tumor markers (CEA and CA-199) were not elevated. Interestingly, the allele frequency of the 3 CRC-specific mutations in *APC, KRAS* and *TP53* decreased significantly in September 2020. The ctDNA clearance can be related to a response to treatment. The ctDNA status was positive again during follow-up, and his tumor recurrence took place after 9 months later, suggesting that he might carried MRD for a long period. The result confirmed that ctDNA has superior sensitivity and specificity in detecting MRD compared to traditional radiographic or laboratory analyses. Therefore, this case highlights the value of ctDNA monitoring to gain further insight into the evolution of a patient’s response over time.

Another interesting finding of our study was that mutations in *TCF7L2* were tracked by ctDNA analysis (Table S1, Supplemental Digital Content, http://links.lww.com/MD/J272). Recent studies showed that *TCF7L2* gene was associated with liver metastasis and cancer risk in hepatocellular carcinoma.^[9,10]^ Moreover, the loss of TCF7L2 promoted migration and invasion of CRC cells.^[11]^ These data suggested *TCF7L2* was a potential risk factor in mCRC patients with liver metastasis.

This case report reinforced the promising clinical practices of ctDNA analysis in the prognosis, detection of micrometastases and recurrence monitoring in CRC patients.

## Acknowledgments

We owe thanks to the patient in our study and his family members. We acknowledge the staff of Geneplus (Beijing, China) for their assistance to this study.

## Author contributions

**Conceptualization:** Jiamin Luo, Bo Zhou.

**Data curation:** Jiamin Luo, Jinming Zhou.

**Investigation:** Jing Yuan.

**Methodology:** Liang Zhao, Lu Shen.

**Software:** Liang Zhao, Lu Shen.

**Validation:** Liang Zhao, Lu Shen.

**Writing – original draft:** Jiamin Luo, Bo Zhou.

**Writing – review & editing:** Fang Li.

**Supervision:** Chengyuan Qian.

## Supplementary Material


