# Supplementary material for: Circulating tumor DNA analysis detects micrometastatic disease and predicts recurrence in a patient with colon cancer: A case report
Source: Medicine (Baltimore). 2023 Jul 14;102(28):e34330. doi: 10.1097/MD.0000000000034330 (PMC10344479; doi:10.1097/MD.0000000000034330)
Supplement: Supplementary file 1 [file medi-102-e34330-s001.pdf]

Supplementary Table S1 The ctDNA analysis during treatment in CRC patient with small lung nodules

Gene	cHGVS	pHGVS	Variant allele frequency					
			Baseline 2019-10-23	Post-operation 2019-12-26	Resistance to Chemotherapy 2020-04-03	1 month after Radiofrequency ablation (CR) 2020-09-27	10 months after Radiofrequency ablation 2021-06-21	13 months after Radiofrequency ablation 2021-09-24
			Colorectal Tumor	Plasma ctDNA	Plasma ctDNA	Plasma ctDNA	Plasma ctDNA	Plasma ctDNA
TCF7L2	c.1395A[9>10]	p.C469Vfs*8	12.1%	2.1%	1.8%	ND	ND	ND
ASXL1	c.2893C>T	p.R965*	36.6%	1.3%	0.2%	ND	ND	ND
TCF7L2	c.1343G>A	p.R448Q	24.8%	0.7%	0.2%	ND	ND	2.6%
APC	c.2211C>A	p.Y737*	23.5%	1.0%	0.2%	ND	0.1%	2.5%
TP53	c.329G>T	p.R110L	33.7%	0.7%	0.2%	ND	0.2%	3.3%
KRAS	c.35G>T	p.G12V	29.9%	0.8%	0.1%	ND	ND	ND
BRCA2	c.8242G>A	p.G2748S	2.9%	0.7%	0.1%	ND	ND	1.4%
MGA	c.7396C>G	p.R2466G	28.0%	0.9%	ND	ND	ND	1.1%
ZFP36L2	c.332C[3>4]	p.L112Pfs*14	22.8%	1.4%	ND	ND	ND	ND
APC	c.4464_4465dupAT	p.L1489Yfs*19	20.0%	0.6%	ND	ND	ND	1.0%
KDR	c.3064C>T	p.R1022*	11.7%	0.3%	ND	ND	ND	1.3%
LRP1B	c.3146C>A	p.S1049Y	9.4%	0.5%	ND	ND	ND	1.4%
SLCO1B3	c.734T>C	p.I245T	5.0%	ND	ND	ND	ND	ND
APC	c.3018_3019delTAins GT	p.H1006_K1007delinsQ*	3.3%	ND	ND	ND	ND	ND

CRNKL1	c.333_335delAGCinsT GT	p.Q111_A112delinsHV	2.2%	ND	ND	ND	ND	ND

ND, not detected. HGVS, Human Genome Variation Society
